# Applications of Optical Coherence Tomography in the Diagnosis of Enamel Defects

**DOI:** 10.3390/diagnostics12030636

**Published:** 2022-03-05

**Authors:** Mihai Popescu, Monica Scrieciu, Eugen Osiac, Marilena Bătăiosu, Diana Vlăduțu, Roxana Maria Pascu, Andreea Stănuși, Adina Dorina Glodeanu, Mihaela Ionescu, Veronica Mercuț

**Affiliations:** 1 Department of Pedodontics, University of Medicine and Pharmacy of Craiova, 200349 Craiova, Romania; mihai_15005@yahoo.com (M.P.); marilena.bataiosu@umfcv.ro (M.B.); 2 Department of Prosthodontics, University of Medicine and Pharmacy of Craiova, 200349 Craiova, Romania; dianavladutu04@gmail.com (D.V.); pascuroxana81@yahoo.com (R.M.P.); andreeacazan22@gmail.com (A.S.); veronica.mercut@umfcv.ro (V.M.); 3 Department of Biophysics, University of Medicine and Pharmacy of Craiova, 200349 Craiova, Romania; eugen.osiac@umfcv.ro; 4 Department of Internal Medicine, University of Medicine and Pharmacy of Craiova, 200349 Craiova, Romania; glodeanuadina@yahoo.com; 5 Department of Medical Informatics and Biostatistics, University of Medicine and Pharmacy of Craiova, 200349 Craiova, Romania

**Keywords:** developmental defects of enamel, optical coherence tomography, hypomineralization, hypoplasia

## Abstract

Developmental defects of enamel (DDEs) are deviations from the normal appearance in terms of the quantity and quality of tooth enamel. They may be genetic or acquired. The most important DDEs are hypomineralization and hypoplasia. The aim of this study was to produce “in vivo” DDE in Wistar rats by administering amoxicillin to pregnant females and to highlight these lesions after sacrifice of the pups by macroscopic and microscopic examination and optical coherence tomography (OCT). Amoxicillin (100 mg/kg) was administered to two pregnant Wistar female rats for the production of DDEs. When the pups were 2 months old, they were sacrificed, and their jaws were harvested together with their teeth. The jaws were examined macroscopically, microscopically, and by OCT. Following the macroscopic and microscopic examination, it was established that four pups had a total of 42 DDE lesions. At the OCT examination, the hypomineralization was characterized by an intense, inhomogeneous OCT signal, and the hypoplasia was characterized by the absence of the signal. Administration of amoxicillin to pregnant females of Wistar rats resulted in DDEs in their offspring. The OCT examination confirmed the presence of these lesions in the teeth of rat pups.

## 1. Introduction

Developmental defects of dental enamel (DDEs) are deviations from the normal appearance in terms of the quantity and quality of tooth enamel, due to a disturbance during embryogenesis. The dental enamel defects are classified into two major categories: genetic DDEs, which occur in very rare general syndromes (1.4/1000 to 1/15,000 cases, depending on the number of population studied) [1], or acquired DDEs, which are much more frequent, with manifestations strictly at the level of the teeth [2].

Acquired enamel defects are classified into hypoplasia [3,4], hypomineralization [5], fluorosis [6], and intrinsic dyschromia [7].

The importance of these diseases is given by the high prevalence, increasing, with values of 11.22% [8], 11.27% [9], 29.9% [10], and 33.7% in children aged 5 years [11], the associated complications and therapeutic difficulties encountered in both temporary and permanent dentition [12].

Acquired enamel defects have a multifactorial etiology related to genetic predisposition, perinatal hypoxia, prematurity, and some diseases of the infant and young child [12], and they are associated with the use of antibiotics in the first years of the child’s life [13,14] or by pregnant women [15], especially amoxicillin. Other drugs also represent risk factors for DDE: cefaclor, paracetamol [16], penicillin [17], bronchodilators, corticosteroids [18], antiepileptics [19], celecoxib, erythromycin [20], and tetracycline [21].

In the laboratory, studies in rats allowed the production of DDEs by administering amoxicillin to females during pregnancy [21,22].

These lesions are commonly identified by clinical and radiological examination. Optical coherence tomography (OCT) is an imaging method that offers new perspectives in the diagnosis of dental diseases. OCT can be used “in vitro” or “in vivo”, a situation that allows real-time examination of tissues by section, without the need for biopsy or a histological or radiological examination [23].

The aim of this study was to produce DDEs in the laboratory on Wistar rats by administering amoxicillin to pregnant females, and to establish the utility of 2D OCT in the diagnosis of DDE, as well as the contribution of 3D reconstruction using software applications in the assessment of their disposition and extent.

## 2. Materials and Methods

Two pregnant Wistar female rats were selected for the laboratory production of DDEs, and they were administered 100 mg/kg amoxicillin. After the pups were 2 months old, all 15 pups were sacrificed, and their jaws were examined. The pups were sacrificed according to the current standards, after administration of an anesthetic overdose of Ketamidor 100 mg/mL 20 IU (0.2 mL) and Xilazyn Bio 2% 0.3 mL. The injection was given intraperitoneally, slightly to the right of the abdominal white line.

All stages related to the fertilization of the female rats, the administration of amoxicillin, pups’ sacrifice, the collection of the jaws with the teeth, and the actual examination were performed within the UMF Craiova Biobase.

**Figure 1:**
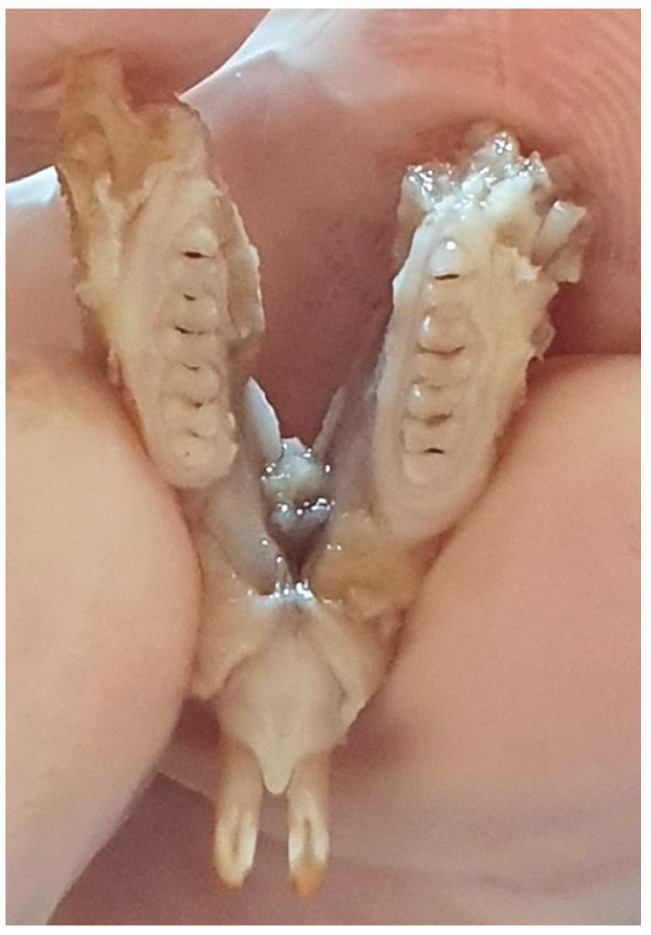
Rat jaw.

After sacrificing the 15 rat pups, the maxilla and mandible of each pup were introduced into 10% formalin until examination. The macroscopic examination was performed using a consultation kit, air spray, the light source from the unit, and a magnifying glass with a diameter of 90 mm (Figure 1).

Due to the small size of the rat teeth, after the macroscopic examination and identification of the enamel defects, examination by optical microscopy was performed to confirm the diagnosis of enamel defects. A Leica DM2500 microscope was used, with a 4× objective, and a 10× objective for obtaining detailed images.

The OCT examination was performed using an OCT OCS 1300 SS device produced by Thorlabs. The OCT device has a laser source with a center wavelength of 1310 nm, a spectral bandwidth of 100 nm, and an average power of 12 mW.

Prior to the OCT examination, the jaws with the teeth were removed from the formalin containers, washed with saline solution, and dried with paper towels. Each piece was fixed in high-strength “Zetaplus Zhermack” silicone to ensure a perpendicular projection of the light beam on the examined surface. Two-dimensional scans of rat teeth surfaces were performed. The OCT device had an axial resolution of 12 μm and a side resolution of 15 μm, which allowed the investigation in 30 s of a sample of 10 mm × 10 mm × 3 mm (length, width, depth) or 1024 pixels × 1024 pixels × 512 pixels. In this study, a design was used to examine the dental surfaces from several angles so that the dental developmental defects could be diagnosed.

To better visualize the magnitude and orientation of the analyzed enamel defects, OCT 2D images were processed using ImageJ in 3D images.

ImageJ is an image processing program proposed by the National Institutes of Health and the Laboratory for Optical and Computational Instrumentation (University of Wisconsin) in 1997.

The study was approved by the Ethics Commission of University of Medicine and Farmacy in Craiova, Decision no. 7 of 20 January 2021, by the Animal Welfare Commission, Decision no. 210 of 29 October 2020, and by the Project Authorization no. 10 of 3 March 2021, issued by the Sanitary Veterinary and Food Safety Directorate in Dolj County.

## 3. Results

Of the 15 pups sacrificed, following the macroscopic examination completed with the examination by light microscopy, four pups (representing 26.66%) presented DDEs.

For these four pups, the analysis of all 64 teeth showed a total of 42 teeth (65.62%) with enamel defects located at the surfaces of the frontal and lateral teeth, of which nine (representing 21.43% of the total enamel defects) were classified as hypoplasia and 33 (representing 78.57%) were classified as hypomineralization (Table 1).

**Figure 2:**
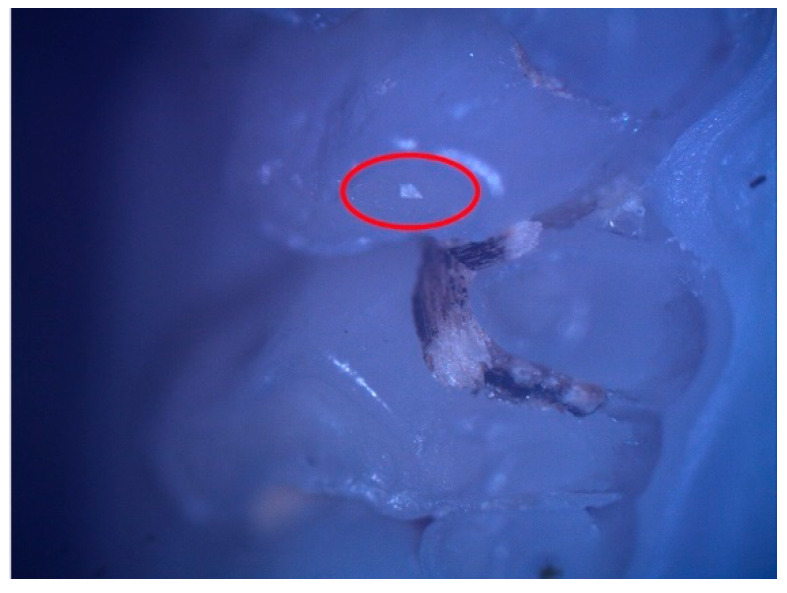
Hypomineralization lesion (surrounded by the red oval) on the occlusal surface of 26.

**Figure 3:**
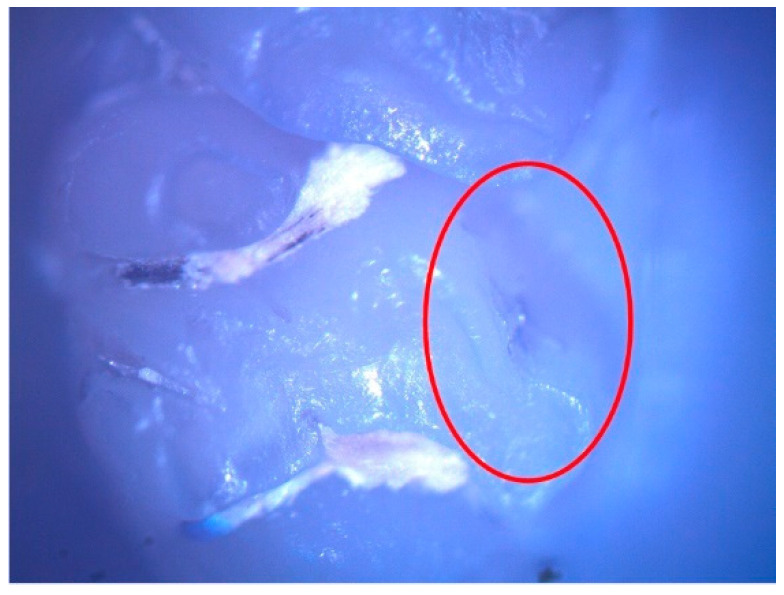
Hypoplasia lesion (surrounded by the red oval) on the palatal surface of 27.

These enamel defects can be described macroscopically as small translucent spots on the tooth enamel for hypomineralization and discontinuities of the tooth enamel for hypoplasia. The microscopic examination confirmed the macroscopic diagnosis (Figure 2 and Figure 3).

After establishing the diagnosis of DDE, the eight arches were examined by OCT, in the 64 teeth belonging to the four pups.

**Figure 4:**
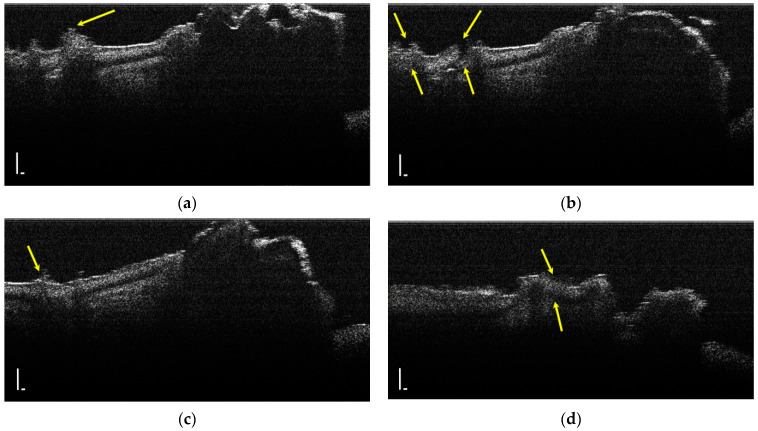
Two-dimensional OCT images of a tooth with hypomineralization (areas marked by yellow arrows): (**a**) cusp presenting a hypomineralization area; (**b**) extended area of hypomineralization, divided by a bridge of healthy enamel; (**c**) small-size hypomineralization area; (**d**) cusp presenting a hypomineralization area (scale bar 100 μm).

During the OCT examination of enamel defects, the hypomineralization was characterized by an intense, inhomogeneous OCT signal. In those areas, the enamel was demineralized; therefore, the image was diffuse (Figure 4).

**Figure 5:**
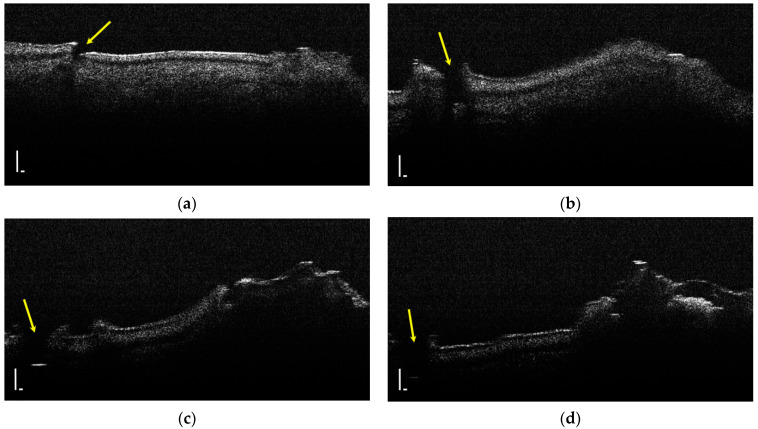
Two-dimensional OCT images of a tooth with hypoplasia (areas marked by yellow arrows): (**a**–**d**) four frames emphasizing the same hypoplasia lesion, from different perspectives (scale bar 100 μm).

Hypoplasia represents a lack of substance and a discontinuity of the tooth enamel; thus, it was identified by the lack of an OCT signal (Figure 5).

**Figure 6:**
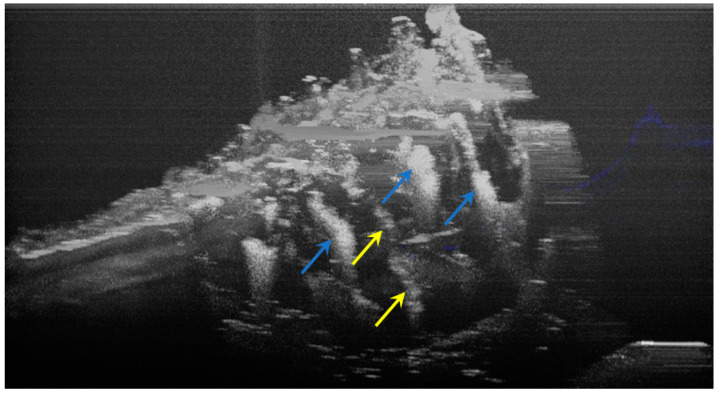
Three-dimensional OCT reconstruction of hypomineralization areas (Axis 0Y). The blue arrows indicate three well-defined areas; the yellow arrows indicate several small-size diffuse areas.

**Figure 7:**
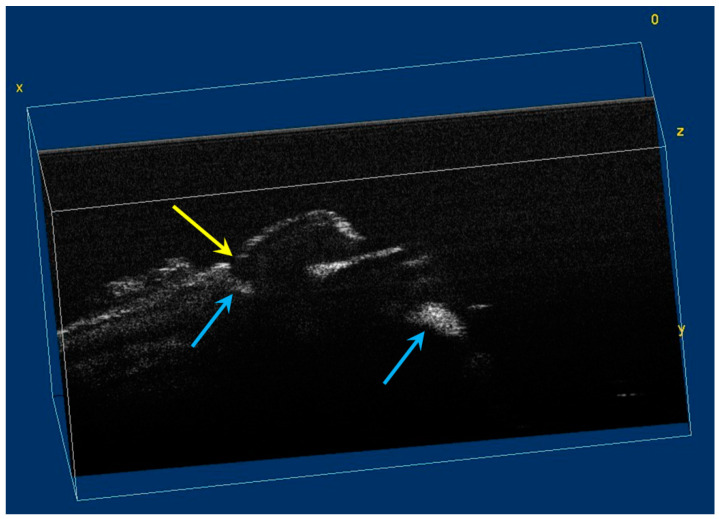
Volumetric reconstruction (in ImageJ) of hypomineralization areas marked by blue arrows. The yellow arrow indicates a small hypoplasia lesion, which was not initially identified in 2D OCT images analysis.

**Figure 8:**
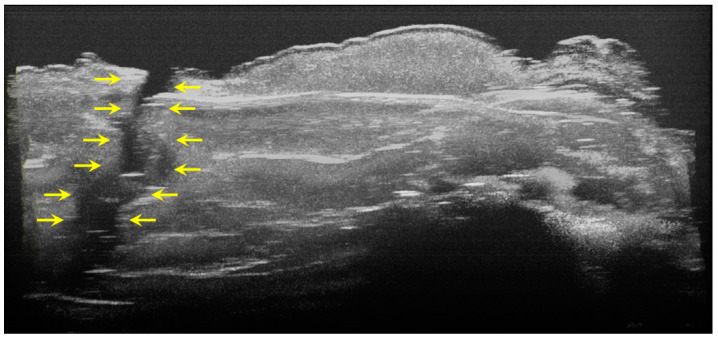
Three-dimensional OCT reconstruction (in ImageJ) of a hypoplasia lesion (Axis 0Y). The yellow arrows indicate a continuous area characterized by the absence of signal, similar to the appearance of a dental crack or fracture.

**Figure 9:**
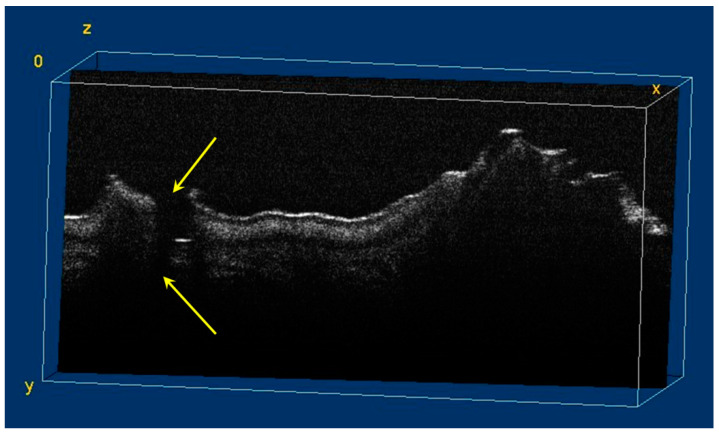
Volumetric reconstruction (in ImageJ) of a hypoplasia lesion. The yellow arrows indicate the affected area, defined by a discontinuity of the enamel at the surface of the tooth; this lesion extends beyond the dentinoenamel junction.

The image processing using ImageJ software showed the spatial disposition and extent of the hypomineralization lesions and hypoplasia (Figure 6, Figure 7, Figure 8 and Figure 9).

## 4. Discussion

DDEs are currently a topic of great importance in the specialty literature. They produce physiognomic disorders and dental sensitivity; they are associated with a high risk of carious damage and a predisposition to erosion [24,25].

In 2019, Andrade found that oral manifestations can even affect the quality of life [11]. The management of patients with enamel defects should aim to prevent these injuries and treat them as early as possible in order to prevent complications and laborious treatments that, in some cases, may lead to the sacrificing of the pulpal organ and the need to apply crowns over the affected teeth or even tooth extraction in severe cases [12,26,27]. Under these conditions, the prevention aims to eliminate all etiological factors that may act in the uterine life or immediately after birth, early detection of these lesions, and establishing the extension for choosing the best therapeutic option. However, the clinical examination does not provide enough information. Conventional radiological examination and high-resolution 3D radiological techniques (cone beam computed tomography (CBCT)) involve a high dose of radiation, which is not justified for DDE treatment [28,29].

In this context, the present study describes the applications of the OCT in the diagnosis of DDE. A nonpolarized OCT system was used, which provided images for each dental defect. Each scan included a total of 512 images, a significant amount of data to help establish the diagnosis.

OCT is an effective diagnosis technique that does not expose the patient to ionizing radiation [28]; the method can also be used for children and pregnant women [30]. The OCT technique uses an infrared laser [31] to investigate biological structures at a depth of up to 2–3 mm, depending on the light source and the dispersion properties of the analyzed structure [32]. The first in vitro and in vivo OCT images of hard and soft dental tissues were obtained by Colston and colleagues [33]. Since then, OCT has become a very popular method of investigation in dentistry, used to investigate tooth tissues, such as caries, hypomineralization and remineralization of enamel and dentin [34,35,36], periodontal lesions, and even oral cancer [37].

More recently, OCT was used to investigate temporary teeth with dental wear [38], permanent teeth with dental wear [39], and even dentin remineralization [40].

In this study, Wistar rat teeth with DDEs produced after amoxicillin administration in the uterine life were examined. Studies to produce DDEs in rats following amoxicillin administration to pregnant females have also been performed [21,22]. The use of OCT for DDE diagnosis has several advantages over the clinical or radiological method. OCT allows the visualization of the internal structure of the enamel at a high spatial resolution (12 μm); the details of these images can be obtained only microscopically [28,41].

The interface between the air (the dark area above the enamel) and the enamel appears bright due to the high dispersion of the incident photons, which encounter a region with a high refractive index and an irregular surface. Subtle variations in the gray contrast of the image may also show small variations in the mineral content of the enamel.

The hypomineralized enamel and dentin appear bright on the OCT image, due to the high dispersion signal [30]. This dispersion at the level of the mineral deficiency zone occurs as a result of the formation of numerous microporosities inside the structure in which the variation of the local refractive index took place. Hariri and colleagues measured the refractive indices of hypomineralized and remineralized enamel and dentin and demonstrated the direct proportional relationship between mineral density and refractive indices [42]. Thus, the high dispersion OCT signal is the diagnostic criterion for hypomineralization [30]. On the OCT image, hypomineralization appears white on a gray background, by increasing the dispersion signal [42]. This is in contrast to the radiolucent appearance of hypomineralization on the radiological examinations [43,44]. Therefore, OCT allows the clear identification of the hypomineralized enamel even at the early stage [30], as well as the extent of the defect, both in area and in depth [44].

DDEs are identified on the OCT image by the high light dispersion, due to the higher porosity and the depth, with the severity of the defects being implicitly measured in the same way [45]. The severity of hypomineralization can also be assessed on the OCT images by measuring the loss of penetration depth [46,47,48]. The previous methods used to evaluate DDEs were qualitative, based on tooth color and morphology. The ability to quantify the depth and severity of DDEs brings great benefits in terms of therapeutic conduct. OCT also helps to differentiate the hypomineralization from the incipient demineralization existing in the case of a carious lesion, according to DDEs having a more uniform geometry and being spread over a larger area [45].

Polarization-sensitive optical coherence tomography (PS-OCT) is a step forward in the evolution of OCT technologies [49].

PS-OCT also allows the examination of the DDE tooth structure, including the surface layer with a higher mineral content [34]. This is very important because it helps to differentiate inactive DDEs that have stopped evolving from active and evolving DDEs [45]. Although PS-OCT has the ability to highlight DDE including at the dentin level, which allows the assessment of the severity of the defect by integrating the reflectivity with depth, the interpretation is quite difficult, and the scan does not cover the entire tooth surface. However, these shortcomings have been corrected by introducing domain frequency OCT, a method capable of providing real-time 3D images with very good resolution on very large areas of the tooth [45].

Following the use of PS-OCT, hypomineralization differentiates itself from healthy tissue by increasing reflectivity and changing the birefringence of the affected enamel [50]. This was confirmed by Jones and Fried, who examined teeth with artificial carious lesions, which underwent remineralization, using PS-OCT [34]. The authors reported that demineralized surfaces that were exposed for 20 days to a fluorinated solution for remineralization had similar reflectivity to healthy enamel. Thus, the increase in the mineral volume led to a significant decrease in optical reflectivity over a large area.

“In vivo” studies have also been performed showing that PS-OCT is a minimally invasive optical method that can be used to clinically assess the severity of hypomineralization by providing high-quality images [51]. The goal was achieved in the case of buccal hypomineralization and especially occlusal hypomineralization. The topography of occlusal surfaces is an advantage in the OCT examination, as there is a lower risk of producing a strong specular reflection, as in the case of smooth surfaces. Thus, in case of flat dental surfaces, artefacts can be generated, which make it difficult to visualize the superficial hypomineralization.

In the presented study, the lesions at the level of the occlusal and buccal surfaces varied in position and severity, which increased their difficulty in interpretation. Moreover, in the case of extensive defects, there is a loss of intensity in depth and then the dentinoenamel junction can no longer be identified. The solution for these situations is to obtain 3D tomographic images, covering the entire tooth surface [51]. Unfortunately, the device used did not allow obtaining 3D images, but only 2D images. However, the use of the ImageJ program led to the 3D reconstruction of the investigated lesions, which allowed a more accurate assessment of the extent (surface and depth), of the orientation of the lesions, and even of the dentinoenamel junction. Software applications dedicated to image processing are widely used in medicine, to increase the accuracy of 2D image content detection, or to reconstruct volumetric elements for detailed analysis [52,53,54]. For our study, the 3D reconstruction presented multiple advantages: the possibility to evaluate a specific lesion within a volumetric image (analysis on all 3 axes); the possibility to determine the spatial disposition and extent of a specific lesion; an increased level of accuracy regarding the identification of small lesions that may be missed following single 2D image analysis.

In addition to the use of the OCT for the diagnosis and evaluation of the DDE size, the method also allows the evaluation of the effectiveness of the treatment, by highlighting the formation of a highly mineralized surface layer with low reflectivity. The OCT can also be used before initiating treatment to identify a mineralized layer that indicates the existence of an inactive defect, which can be monitored [34].

This study had several limitations. The small number of rat pups who developed DDEs led to a relatively reduced number of enamel lesions, and their analysis was rather difficult considering the small size of each tooth. Furthermore, due to the differences in the morphostructural features of rat and human teeth, the OCT images cannot be considered fully comparable. However, this study allowed us to describe certain patterns of hypoplasia and hypomineralization.

## 5. Conclusions

The administration of amoxicillin to pregnant Wistar rats allowed the production of DDEs in rat pups in the form of hypomineralization and hypoplasia. DDE lesions were detected by macroscopic examination and microscopic examination. The 2D OCT examination allowed highlighting the lesions of hypomineralization by an intense inhomogeneous signal and those of hypoplasia by the absence of the signal. The disposition and extent of the lesions could also be determined using the ImageJ software application. Our study confirms the applicability of the 2D OCT examination technique in DDE evaluation, while the subsequent 3D reconstruction increased the assessment level of our results.

## Figures and Tables

**Table 1:** Distribution of our study lot, according to DDE presence.

	Rat Pups	
	with DDE	without DDE
Number of pups	4	11
Number of analyzed teeth	64	484
Number of DDE	42	0
Hypoplasia	9	0
Hypomineralization	33	0

## Data Availability

The authors declare that the data of this research are available from the corresponding authors upon reasonable request.
